# Enhanced replication of SARS-CoV-2 Omicron BA.2 in human forebrain and midbrain organoids

**DOI:** 10.1038/s41392-022-01241-2

**Published:** 2022-11-20

**Authors:** Yuxin Hou, Chang Li, Chaemin Yoon, On Wah Leung, Sikun You, Xiaoming Cui, Jasper Fuk-Woo Chan, Duanqing Pei, Hoi Hung Cheung, Hin Chu

**Affiliations:** 1 State Key Laboratory of Emerging Infectious Diseases, Department of Microbiology, School of Clinical Medicine, Li Ka Shing Faculty of Medicine, The University of Hong Kong, and Centre for Virology, Vaccinology and Therapeutics, Hong Kong Science and Technology Park, Hong Kong Special Administrative Region, China; 2 School of Biomedical Sciences, Faculty of Medicine, and Institute for Tissue Engineering and Regenerative Medicine (iTERM), The Chinese University of Hong Kong, Hong Kong Special Administrative Region, China; 3 Centre for Regenerative Medicine and Health, Hong Kong Institute of Science and Innovation, Chinese Academy of Sciences, Hong Kong Special Administrative Region, China

**Keywords:** Microbiology, Embryonic stem cells

**Dear Editor,**

Coronavirus Disease 2019 (COVID-19) is associated with a variety of neurological complications, including encephalopathy, encephalitis, dementia, and others.^1^ The pathogenic mechanism of these neurological manifestations remains incompletely understood but may be due to factors such as coagulation problem, immune-mediated response, or direct viral invasion into the central nervous system (CNS).^2^ We and others previously reported that ancestral SARS-CoV-2 could infect and replicate in human brain organoids.^3,4^ More recently, SARS-CoV-2 Omicron BA.1 emerged in late 2021 and demonstrated altered virological features including increased immunoevasion and attenuated pathogenicity comparing to SARS-CoV-2 wildtype (WT) and previous variants.^5^ However, the neuroinvasiveness of Omicron sublineages remain unexplored. Here, we investigated the neuroinvasion and neurotoxicity of Omicron BA.1 and BA.2, and compared the findings with those of SARS-CoV-2 WT and Delta in human forebrain and midbrain organoids. Our results demonstrated that BA.2 replicated more efficiently while triggered lower levels of type I interferon response than that of SARS-CoV-2 WT, Delta, and BA.1 in both human forebrain and midbrain organoids. In addition, BA.2 triggered substantially higher levels of apoptosis in the infected human forebrain and midbrain organoids. Together, these findings suggest that BA.2 may be different from SARS-CoV-2 WT and previous variants in its capacity in targeting and causing diseases in the human brain.

To model the susceptibility of human brain cells to different SARS-CoV-2 variants, we established forebrain and midbrain organoids from human embryonic stem cells following previously described protocols (Fig. 1a, b).^6,7^ Forebrain organoids mimic human cerebral cortex development and contain dorsal telencephalic tissue after 3 weeks of differentiation. Stratified neuroepithelial structures express neural stem/progenitor cell (NSC/NPC) markers (SOX2 and NESTIN) and forebrain marker PAX6 after spatial patterning (day 9), and mature cortical neuron markers CTIP2 and SATB2 after 30 days of differentiation (Fig. 1c). Midbrain organoids mimic mesencephalic neurogenesis and express midbrain dopaminergic (mDA) progenitor markers (FOXA2 and LMX1A) between day 9 and 19. Midbrain Organoids at day 30 started to express mature mDA neuronal markers (TH and DAT) (Fig. 1c). Quantitative PCR analysis of additional markers confirmed the forebrain and midbrain identities of the generated organoids (Fig. 1d). Both day 30 forebrain and midbrain organoids expressed more ACE2 and endosomal entry proteases (cathepsin B and cathepsin L), while expressed less plasma membrane entry protease (TMPRSS2), compared to their day 0 counterparts (Supplementary Fig. 1a).

**Fig. 1:**
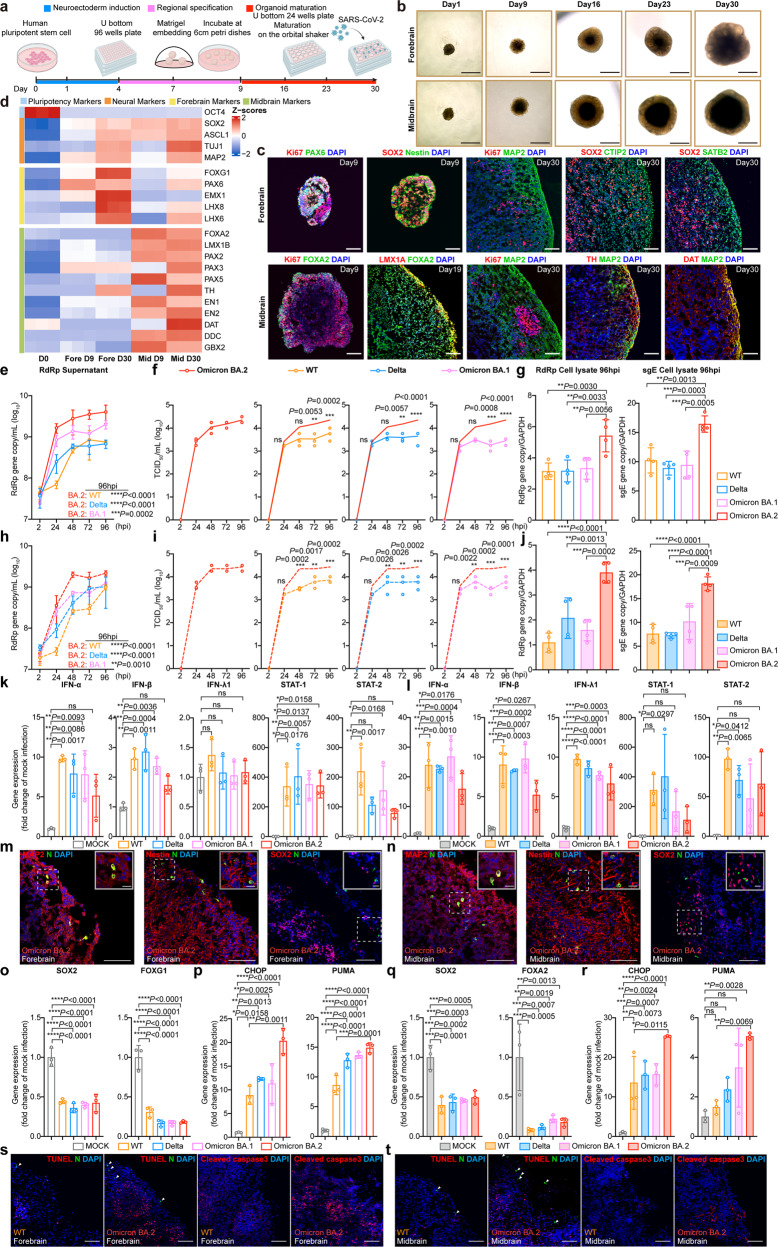
Virological features of SARS-CoV-2 WT, Delta, Omicron BA.1 and Omicron BA.2 in human forebrain and midbrain organoids. **a**–**d** Schematic diagram of human embryonic stem cells (H1)-derived brain organoids generation. H1 was purchased from WiCell Research Institute. Formation of brain organoids were achieved by a series of protocol that included neuroectoderm induction, regional specification, and organoid maturation. Brain organoids were infected by SARS-CoV-2 after day 30 (**a**). Representative bright-field images of forebrain and midbrain organoids at day 1, 9, 16, 23, and 30. Scale bars represent 1 mm (**b**). Characterization of forebrain organoids (9- and 30-day-old) and midbrain organoids (9-, 19-, and 30-day-old). Forebrain organoids were immunostained with Ki67, PAX6, SOX2, or Nestin at day 9 and Ki67, SOX2, MAP2, CTIP2, or SATB2 at day 30. Midbrain organoids were immunostained with Ki67 and FOXA2 at day 9, LMX1A and FOXA2 at day 19, and Ki67, MAP2, TH, or DAT at day 30. Scale bars represent 100 µm (**c**). Heatmaps representing the pluripotency gene markers, neural gene markers, forebrain gene markers, and midbrain gene markers enriched in brain organoids. Forebrain and midbrain organoids were collected at days 0, 9, and 30. Gene expression was detected by RT-qPCR. Z score represents the log10-transformed value (**d**). **e**–**g** Virus replication of H1-derived forebrain organoids. Forebrain organoids were challenged with 3 × 10^5^ PFU of SARS-CoV-2 WT, Delta, Omicron BA.1 and Omicron BA.2. Viral supernatant samples were harvested at the designated time points for quantification of RdRp gene of SARS-CoV-2 by RT-qPCR (*n* = 4) (**e**). Infectious virus titer was determined by median tissue culture infectious dose (TCID_50_) assays in VeroE6-TMPRSS2 cells (*n* = 3). The same set of Omicron BA.2 TCID_50_ result was used to conduct comparisons (**f**). Virus gene copies in cell lysates at 96 hpi were quantified with RT-qPCR against SARS-CoV-2 RdRp gene and subgenomic RNA of the envelope gene (sgE) (*n* = 4) (**g**). Statistical significance between groups was determined with one-way ANOVA with Dunnett’s tests (**g**), two-way ANOVA with Dunnett’s tests (**e**), or two-way ANOVA with Šidák’s tests (**f**). **h**–**j** Virus replication of human embryonic stem cells-derived midbrain organoids. Midbrain organoids were challenged with 3 × 10^5^ PFU of SARS-CoV-2 WT, Delta, Omicron BA.1 and Omicron BA.2. Viral supernatant samples were harvested at the designated time points for quantification of RdRp gene of SARS-CoV-2 by RT-qPCR (*n* = 3) (**h**). Infectious virus titer was determined by TCID_50_ assays in VeroE6-TMPRSS2 cells (*n* = 3). The same set of Omicron BA.2 TCID_50_ result was used to conduct comparisons (**i**). Virus gene copies in cell lysates at 96 hpi were quantified with RT-qPCR against SARS-CoV-2 RdRp gene and sgE (*n* = 4) (**j**). Statistical significance between groups was determined with one-way ANOVA with Dunnett’s tests (**j**), two-way ANOVA with Dunnett’s tests (**h**), or two-way ANOVA with Šidák’s tests (**i**). **k** SARS-CoV-2-infected or mock-infected forebrain organoids were collected at 96 hpi. Host gene expression was quantified with RT-qPCR (*n* = 3). Statistical significance between groups was determined with one-way ANOVA with Dunnett’s tests. **l** SARS-CoV-2-infected or mock-infected midbrain organoids were collected at 96 hpi. Host gene expression was quantified with RT-qPCR (*n* = 3). Statistical significance between groups was determined with one-way ANOVA with Dunnett’s tests. **m** Omicron BA.2-infected forebrain organoids were collected at 96 hpi. Colocalization between viral N protein and cellular markers (MAP2, Nestin, SOX2) was determined with immunostaining. Scale bar represents 100 μm. Scale bar in the insets represents 20 μm. **n** Omicron BA.2-infected midbrain organoids were collected at 96 hpi. Colocalization between viral N protein and cellular markers (MAP2, Nestin, SOX2) was determined with immunostaining. Scale bar represents 100 μm. Scale bar in the insets represents 20 μm. **o**, **p** SARS-CoV-2-infected or mock-infected forebrain organoids were collected at 96 hpi. Expression of neural markers (SOX2 and FOXG1) (**o**) or apoptosis markers (CHOP and PUMA) (**p**) were quantified with RT-qPCR (*n* = 3). Statistical significance between groups was determined with one-way ANOVA with Dunnett’s tests (**o**) or one-way ANOVA with Tukey’s tests (**p**). **q**, **r** SARS-CoV-2-infected or mock-infected midbrain organoids were collected at 96 hpi. Expression of neuronal markers (SOX2 and FOXA2) (**q**) or apoptosis markers (CHOP and PUMA) (**r**) were quantified with RT-qPCR (*n* = 3). Statistical significance between groups was determined with one-way ANOVA with Dunnett’s tests (**q**) or one-way ANOVA with Tukey’s tests (**r**). **s** SARS-CoV-2 WT- or Omicron BA.2-infected forebrain organoids were collected at 96 hpi. TUNEL and cleaved caspase-3 staining were performed. Scale bar represents 100 μm. **t** SARS-CoV-2 WT- or Omicron BA.2-infected midbrain organoids were collected at 96 hpi. TUNEL and cleaved caspase-3 staining were performed. Scale bar represents 100 μm. Data represented mean and standard deviations from the indicated number of biological repeats. Statistical significance between groups was determined with one-way ANOVA (**g**, **j**–**l** and **o**–**r**) or two-way ANOVA (**e**, **f**, **h**, **i**). *p < 0.05, **p < 0.01, ***p < 0.001, ****p < 0.0001. ns not significant

Next, we challenged day 30 forebrain and midbrain organoids with SARS-CoV-2 for virological assessments. We found that BA.2 replicated more efficiently in forebrain organoids than that of SARS-CoV-2 WT, Delta, and BA.1. At 96 hpi, significantly more viral RdRp gene was detected in the culture supernatant of BA.2-infected forebrain organoids than SARS-CoV-2 WT-, Delta-, and BA.1-infected forebrain organoids (Fig. 1e). The infectious virus titer generated from BA.2-infected forebrain organoids at 96 hpi was 3.7- (*p* = 0.0002), 5.1- (*p* < 0.0001), and 7.9-folds (*p* < 0.0001) higher than that of SARS-CoV-2 WT-, Delta-, and BA.1-infected samples, respectively (Fig. 1f). In keeping with supernatant results, BA.2 also replicated to higher levels than other evaluated SARS-CoV-2 variants in the cell lysates (Fig. 1g). Similarly, BA.2 also replicated more efficiently than other SARS-CoV-2 variants in the midbrain organoids (Fig. 1h–j), and in the iPSC-derived forebrain/midbrain organoids (Supplementary Fig. 2a, b). The more efficient replication of BA.2 was not due to differential expression of virus entry-related genes upon infection (Supplementary Fig. 1b). To explore the potential mechanism behind the more efficient replication of BA.2, we evaluated the expression of type I (IFN-α and IFN-β) and type III (IFN-λ1) IFN gene expression upon viral challenge. Our results demonstrated that BA.2 induced lower levels of type I IFNs (IFN-α and IFN-β) and pro-inflammatory cytokines (TNF-α and IL-6) than other SARS-CoV-2 variants in forebrain organoids (Fig. 1k and Supplementary Fig. 3). Compared to the mock-infected group, SARS-CoV-2 WT, Delta, BA.1, and BA.2 infection upregulated IFN-α expression by 9.8- (*p* = 0.0017), 7.9- (*p* = 0.0086), 7.8- (*p* = 0.0093), and 5.1-folds (*p* = ns), respectively (Fig. 1k). Next, we assessed IFN expression in midbrain organoids upon SARS-CoV-2 infection and found that BA.2 also triggered the least IFN-α and IFN-β responses (Fig. 1l).

In parallel to the virological assessments, we asked if the neurotropism of BA.2 is different from that of SARS-CoV-2 WT, Delta, and BA.1. To this end, we performed immunostaining for the infected forebrain and midbrain organoids. Our results demonstrated that the nucleocapsid (N) protein of SARS-CoV-2 WT, Delta, BA.1, and BA.2 similarly colocalized with MAP2 and Nestin, but not with SOX2, in both forebrain and midbrain organoids (Fig. 1m, n and Supplementary Fig. 4). Thus, our data indicate that SARS-CoV-2 can target MAP2^+^ mature neurons and Nestin^+^ neural cells but not SOX2^+^ neural stem/progenitor cells. We additionally evaluated the neuron tropism of BA.2 and found that BA.2 is capable of targeting both TH^+^ (DA neuron in midbrain) and TBR1^+^ (cortical neuron in forebrain) neurons, as well as GFAP^+^ gliocytes. In addition, BA.2 infection in brain organoids appeared to result in axon degeneration (Supplementary Fig. 5). Next, we further evaluated the impact of BA.2 infection in the forebrain and midbrain organoids. We found that SARS-CoV-2 infection significantly perturbed the expression of NPC transcription factors (TFs) in the forebrain and midbrain organoids, including SOX2, FOXG1 and FOXA2 (Fig. 1o, q and Supplementary Fig. 6), which may be modulated by the activated IFNs. Importantly, infection of SARS-CoV-2 WT, Delta, BA.1, and BA.2 upregulated the expression of CHOP and PUMA, which are representative pro-apoptotic markers triggered upon highly pathogenic coronavirus infection.^8^ In keeping with its more robust virus replication, BA.2 triggered higher levels of these pro-apoptotic genes in both forebrain and midbrain organoids compared with SARS-CoV-2 WT, Delta, and BA.1 (Fig. 1p, r).^9^ In addition, we evaluated apoptosis induction in the infected forebrain and midbrain organoids with TUNEL and caspase-3 immunostaining. Our results revealed that although the infection of all evaluated SARS-CoV-2 variants induced apoptosis in forebrain and midbrain organoids, BA.2 triggered a substantially higher magnitude of apoptosis compared with that of other SARS-CoV-2 variants (Fig. 1s, t and Supplementary Figs. 7, 8, and 10).

During the revision phase of the study, we additionally evaluated the more recently emerged BA.4.1 and BA.5.2 on their replication in forebrain and midbrain organoids. We found that while BA.4.1 did not replicate as efficiently as BA.2, BA.5.2 replicated to higher levels when compared with BA.2 (Supplementary Fig. 9). The gained virus replication of Omicron sublineages in brain organoids may be of critical medical and public health importance and warrants further investigation.

Together, our study revealed a number of interesting findings which will be important for follow-up studies. First, the reason behind the enhanced replication of BA.2 in brain organoids should be dissected. Second, the importance and mechanism behind the down-regulation of entry-related factors in SARS-CoV-2-infected brain organoids should be investigated. Third, the mechanism of why BA.2 triggered the lowest IFN response in brain organoid deserves further investigation. Forth, the cause and the physiological importance of BA.2-induced apoptosis in the brain organoids should be further dissected.

To date, over 620 million people have been infected by SARS-CoV-2 with a significant proportion of them infected during the Omicron wave. While most patients survived the infection, post-COVID-19 sequela including neurological manifestations are common.^10^ The increased efficiency of BA.2 to replicate and causing apoptosis in the brain organoids is alarming, indicating that the long-term consequence of BA.2 infection in the CNS should be closely monitored.

## Supplementary information

Supplementary materials

## Data Availability

All data used to draw the conclusions in the paper are available upon request.

## Materials availability

The request of experimental materials should be addressed to H.C. (hinchu@hku.hk) (for virological experimental materials) or H.H.C. (cheunghh@cuhk.edu.hk) (for brain organoid experimental materials).
