# Supplementary material for: Enhanced replication of SARS-CoV-2 Omicron BA.2 in human forebrain and midbrain organoids
Source: Signal Transduct Target Ther. 2022 Nov 20;7:381. doi: 10.1038/s41392-022-01241-2 (PMC9676899; doi:10.1038/s41392-022-01241-2)
Supplement: Supplementary file 1 — Supplementary materials [file 41392_2022_1241_MOESM1_ESM.docx]

Supplementary Materials for

Enhanced replication of SARS-CoV-2 Omicron BA.2 in human forebrain and midbrain organoids

Yuxin Hou*, Chang Li*, Chaemin Yoon1*, On Wah Leung, Sikun You, Xiaoming Cui, Jasper Fuk-Woo Chan, Duanqing Pei, Hoi Hung Cheung#, Hin Chu#

Correspondence to: hinchu@hku.hk

**This PDF file includes:**

Materials and Methods

Figures. S1 to S10

Tables S1

Materials and Methods

Cell lines

Human ESC (H1) was purchased from WiCell Research Institute. Human iPSC (iBC) was generated in our laboratory. Both ESC and iPSC were cultured and maintained on matrigel-coated plates with feeder-free medium (mTeSR1 and mTeSR1 PLUS), according to the WiCell protocol. VeroE6-TMPRSS2 cells were maintained in Dulbecco’s Modified Eagle medium (DMEM) supplemented with 10% heat-inactivated fetal bovine serum (FBS), 100 unit/ml penicillin, and 100 μg/ml streptomycin.

Viruses

SARS-CoV-2 wildtype strain HKU-001a (GenBank: MT230904), B.1.617.2 (Delta) (GenBank: OM212471), B.1.1.529.1 (Omicron BA.1) (GenBank: OM212472), B.1.1.529.2 (Omicron BA.2) (GISAID: EPI_ISL_9845731), Omicron BA.4.1 (GISAID: EPI_ISL_13777657) and Omicron BA.5.2 (GISAID: EPI_ISL_13777658) were isolated from patients with laboratory confirmed COVID-19 in Hong Kong. All SARS-CoV-2 strains were cultured and titrated by TCID50 assay using VeroE6-TMPRSS2 cells. All experiments involving infectious SARS-CoV-2 followed approved standard operating procedures of the Biosafety Level 3 facility at Department of Microbiology, HKU.

Antibodies

SARS-CoV-2 nucleocapsid (N) protein was detected by an in-house rabbit immune serum (1:1000) or mouse anti-SARS-CoV-2 N (1:1000) (Sinobioligical, 40588-MM137), or mouse anti-SARS-CoV-2 N (1:1000) (Abcam, #ab281300). Other primary antibodies include mouse anti-Neurofilament 200 (1:400) (Sigma, #SAB4200811), goat anti-GFAP (1:750) (Abcam, #ab4674),

rabbit anti-TBR1 (1:100) (ABclonal, #A19550), goat anti-Chicken Alexa Fluor 488 (Invitrogen, #A32931TR), rabbit anti-SOX2 (1:400) (CST, 23064), mouse anti-SOX2 (1:500) (R&D systems, MAB2018), mouse anti-Nestin (1:500) (CST, 33475), rat Anti-Ctip2 (1:200) (Abcam, ab18465), mouse anti-MAP2 (1:200) (Invitrogen, MA5-12826), mouse anti-PAX6 (1:50) (Abcam, ab245110), rabbit anti-Ki67 (1:300) (Abcam, ab16667)，rabbit anti-MAP2 (1:200) (Abcam, ab96378), mouse anti-SATB2 (1:100) (Santa cruz, 81376), mouse anti-FOXA2 (1:100) (Santa cruz, 101060), mouse anti-LMX1A (1:100) (Abcam, ab139726), rat anti-Dopamine Transporter (1:50) (Sigma, MAB369), mouse anti-TH (1:50) (Santa cruz, 25269), rabbit anti-cleaved caspase 3 (1:500) (CST, 9664). The following secondary antibodies (1:500) were applied for immunofluorescence: goat anti-rabbit Alexa Fluor 488 (Invitrogen, A32731), goat anti-mouse Alexa Fluor 488 (Invitrogen, A32723), goat anti-rabbit Alexa Fluor 647 (Invitrogen, A21244), goat anti-mouse Alexa Fluor 647 (Invitrogen, A32728), goat anti-rat Alexa Fluor 647 (Invitrogen, A21247).

Generation of human brain organoids

Forebrain and midbrain organoids were generated as previously reported. Firstly, human iPSC or hESC were dissociated to single cells with TrypLETM (Gibco). Then, cells were washed and resuspended in mTeSR1 medium and 1x10^4^ single cells were plated in Ultra-low U-bottom 96-well plate (Corning), containing DMEM/F12 (Gibco), supplemented with 20% KSR (Gibco), 1% penicillin/streptomycin (P/S) (Thermo Fisher Scientific), 1% GlutaMAX (Thermo Fisher Scientific), 1% NEAA (Thermo Fisher Scientific), 3% FBS (Gibco), 50 μM Y27632 (Stemgent), 4 ng/ml of bFGF (PeproTech), 55 μM β-mercaptoethanol (Gibco), and 1 μg/ml of heparin (STEMCELL Technologies). After 24 hours, for forebrain organoids generation, the medium was changed to 100 μl brain organoid generation medium (BGM), consisting of 1:1 mix of Neurobasal Medium (Gibco) and DMEM/F12 (Gibco), plus 1% N2 Supplement (Gibco), 2% B27 without vitamin A (Gibco), 1% GlutaMAX, 1% NEAA, 1% P/S, 55 μM β-mercaptoethanol (Gibco), 1 μg/ml heparin, 2 μM Dorsomorphin (Sigma) and 2 μM A83-01 (PeproTech). For midbrain specification, 3 μM CHIR99021 (Tocris) and 1 μM IWP2 (PeproTech) were added in BGM. On day 3, for forebrain, the medium was replaced with 100 μl fresh BGM; for midbrain, the medium was replaced with 100 μl fresh BGM, supplemented with 3 μM CHIR99021 and 1 μM IWP2. On day 4, for forebrain, the medium was changed with 100 μl BGM again. For midbrain, the medium was changed with BGM, supplemented with 100 ng/ml FGF8 (PeproTech), 2 μM SAG (PeproTech), 3 μM CHIR99021 and 1 μM IWP2. On day 5, the medium was changed with 200 μl BGM (the same as day 4). On day 7, organoids were embedded into Matrigel (Corning) and transferred to 6 cm petri dishes containing BGM, plus 200 ng/ml laminin (sigma) and 2.5 μg/ml insulin (Gibco). After incubation in 37℃ for 2 days, embedded organoids were transferred to Ultra-low attachment 24 well plates (Corning) containing brain organoid maturation medium (BMM), which consists of DMEM/F12 supplements with 1% N2 Supplement, 2% B27 without vitamin A, 1% GlutaMAX, 1% NEAA, 1% P/S, 55 μM β-mercaptoethanol, 1 μg/ml heparin, 200 μM ascorbic acid (sigma), 10 ng/ml BDNF (Shenandoah), 10 ng/ml GDNF (Shenandoah), 125 μM dibutyryl-cAMP (PeproTech). Organoids were placed on an orbital shaker in a 37℃ incubator for maturation and the medium was changed every other day with BMM until day 30.

TUNEL assay

TUNEL assay was performed using the DeadEnd™ Fluorometric TUNEL System (Promega) according to manufacturer’s protocol. Briefly, the cryo-sectioned organoids on slides were washed three times in PBS before the TUNEL assay. Samples were permeabilized with permeabilization solution for 1 hour at room temperature and washed once with PBS. Then, 100 μl Equilibration Buffer were added to the samples at room temperature for 10 minutes. The samples were incubated with 50 μl of TdT reaction mix for 1 hour at 37℃. To stop reaction, slides were immersed in 2X saline-sodium citrate (SSC) buffer for 15 minutes and then, washed with PBS for three times. The organoids were blocked in PBS, containing 3% BSA and 5% goat serum at room temperature for 1 hour and co-stained with antibody against SARS-CoV-2 nucleocapsid protein.

SARS-CoV-2 infection

Differentiated brain organoids were sheared mechanically and inoculated with 3x10^5^ PFU WT and variants of SARS-CoV-2 at 37℃. After 2 hours of inoculation, the inoculum was aspirated. The organoids were washed three times with culture medium and further incubated in culture medium. At the indicated time points, the culture medium was harvested for viral titration of extracellular virions using TCID_50_ assay or RT-qPCR against RdRp according to established protocols in the group, whereas organoids were harvested at 96 hpi for the quantification of intracellular viral load and host gene expression, or for immunofluorescence staining.

RNA extraction and quantitative RT-PCR

Organoids were lysed by RLT buffer (Qiagen) and extracted with the RNeasy Mini kit (74106, Qiagen). Supernatant samples were lysed by AVL buffer (Qiagen) and extracted with the QIAamp Viral RNA Mini kit (52906, Qiagen). SARS-CoV-2 replication was detected by RT-qPCR against viral RdRp or sgE by QuantiNova SYBR Green RT-PCR kit (208154, Qiagen) or the QuantiNova Probe RT-PCR Kit (208354, Qiagen). To detect expression level of host genes, samples were performed with reverse transcription using Transcriptor First Strand cDNA Synthesis Kit (Roche) and detected with LightCycler 480 master mix (Roche) using LightCycler 480 Real-Time PCR System (Roche, Basel, Switzerland). All primer and probe sequences are included in **Table S1**.

Immunofluorescence staining

In brief, organoids were fixed overnight in 10% formalin at room temperature. The fixed samples were immersed in 30% sucrose solution and incubated at 4ºC overnight. After sinking to the bottom, organoids were transferred to OCT solution for 1 hour and slowly frozen on dry ice. Frozen blocks were cryo-sectioned at a thickness of 10 µm with CM3050S Cryotome with CryoJane Tape Transfer System (Leica). After washing with PBS, sliced organoids were permeabilized and blocked in PBS with 0.3% Triton, 5% BSA and 10% goat serum for 1 hour at room temperature. Then, samples were stained with primary antibodies diluted in PBS containing 1% BSA and 0.1% triton at 4ºC overnight. After washing with PBS, the sections were incubated with secondary antibodies and DAPI (Sigma) at room temperature for 1 hour. Samples were mounted with ProLong Glass Antifade Mountant (Thermo Fisher Scientific) after several times wash with PBS. Zeiss LSM 880 system (Zeiss) were used for confocal microscopy and images were processed by Zeiss software.

TDID_50_ assay

Infectious titer of SARS-CoV-2 was determined with standard plaque standard TCID_50_ assays. In brief, VeroE6-TMPRSS2 cells were seeded in 96-well plates one day prior to infection. The harvested supernatant samples were 10-fold serially diluted and inoculated to the cells for 1 hour at 37°C. At 72 hpi, virus titer was calculated using the Muench and Reed method.

Quantitative analysis

Quantitative analysis was performed by ImageJ on whole organoid sections. Each analysis consists of 3 organoids per group and 2 or more sections per organoid. DAPI amount was measured by “find maxima”; a corresponding cellular content volonoy mask was generated by "segmented particle". TUNEL speckle was measured directly by “analyze particle”. Threshold fluorescence signal was subtracted by cellular volonoy mask to generate non-fluoresence cellular mask, followed by “analyze particle” to measure fluorescence negative cells.

Statistical analysis

Data represented mean and standard deviations from the indicated number of biological repeats. Statistical comparison between different groups was performed by one-way ANOVA or two-way ANOVA using GraphPad Prism 9. * represented p < 0.05, ** represented p < 0.01, *** represented p < 0.001, **** represented p < 0.0001. ns = not significant.

Figure. S1.


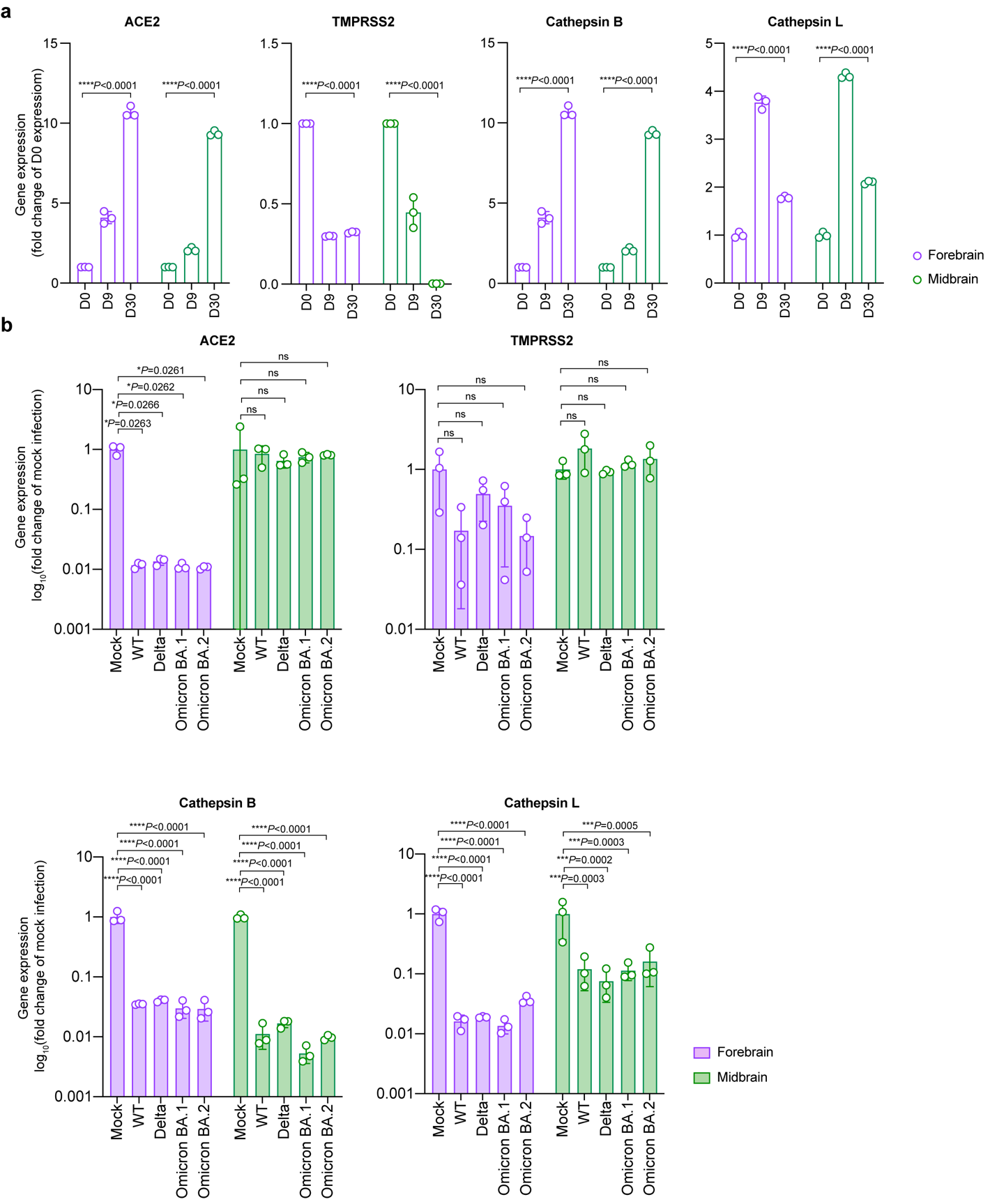
 **Figure. S1. Expression of SARS-CoV-2 entry-related genes. a** Expression of ACE2, TMPRSS2, cathepsin B, and cathepsin L in differentiating forebrain and midbrain organoids were quantified with RT-qPCR (n=3). **b** Expression of ACE2, TMPRSS2, cathepsin B, and cathepsin L in SARS-CoV-2-infected or mock-infected brain organoids was quantified with RT-qPCR (n=3). Data represented mean and standard deviations from the indicated number of biological repeats. Statistical significance between groups was determined with two-way ANOVA with Dunnett’s tests.

Figure. S2.


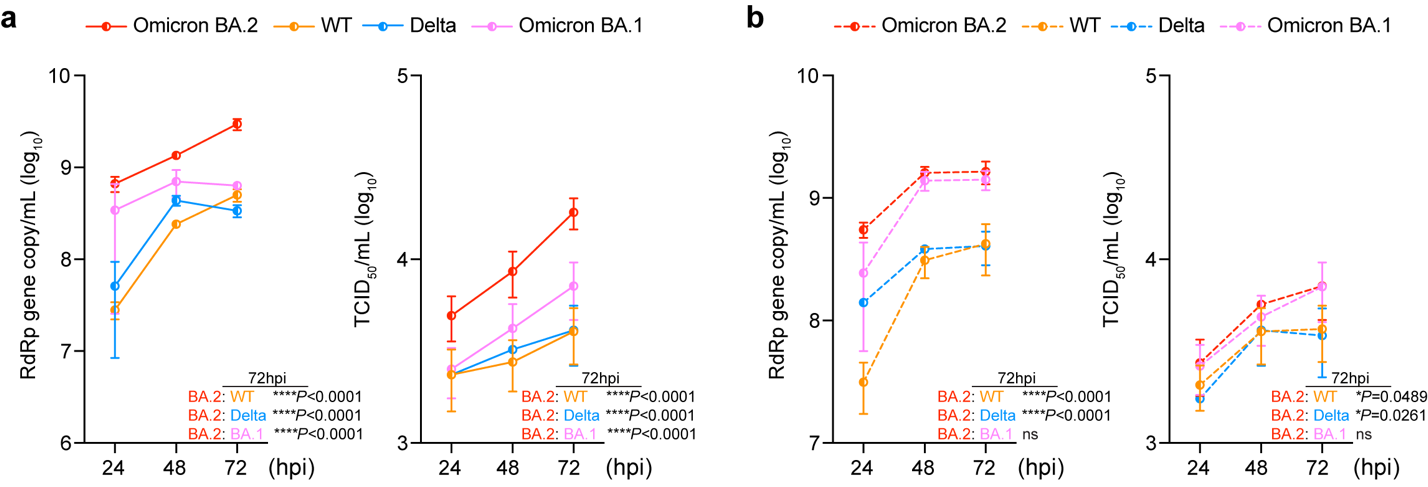
 **Figure. S2. Replication of SARS-CoV-2 wildtype and variants in human iPSC-derived brain organoids. a** Virus replication of iPSC-derived forebrain organoids. Forebrain organoids were challenged with 3x10^5^PFU of SARS-CoV-2 WT, Delta, Omicron BA.1 and Omicron BA.2. Virus replication in supernatants was determined by RT-qPCR against RdRp gene of SARS-CoV-2 (n=3) and TCID_50_ assays (n=3). **b** Virus replication of iPSC-derived midbrain organoids. Midbrain organoids were challenged with 3x10^5^PFU of SARS-CoV-2 WT, Delta, Omicron BA.1 and Omicron BA.2. Virus replication in supernatants was determined by RT-qPCR against RdRp gene of SARS-CoV-2 (n=3) and TCID_50_ assays (n=3). Data represented mean and standard deviations from the indicated number of biological repeats. Statistical significance between groups was determined with two-way ANOVA with Dunnett’s tests.

Figure. S3.


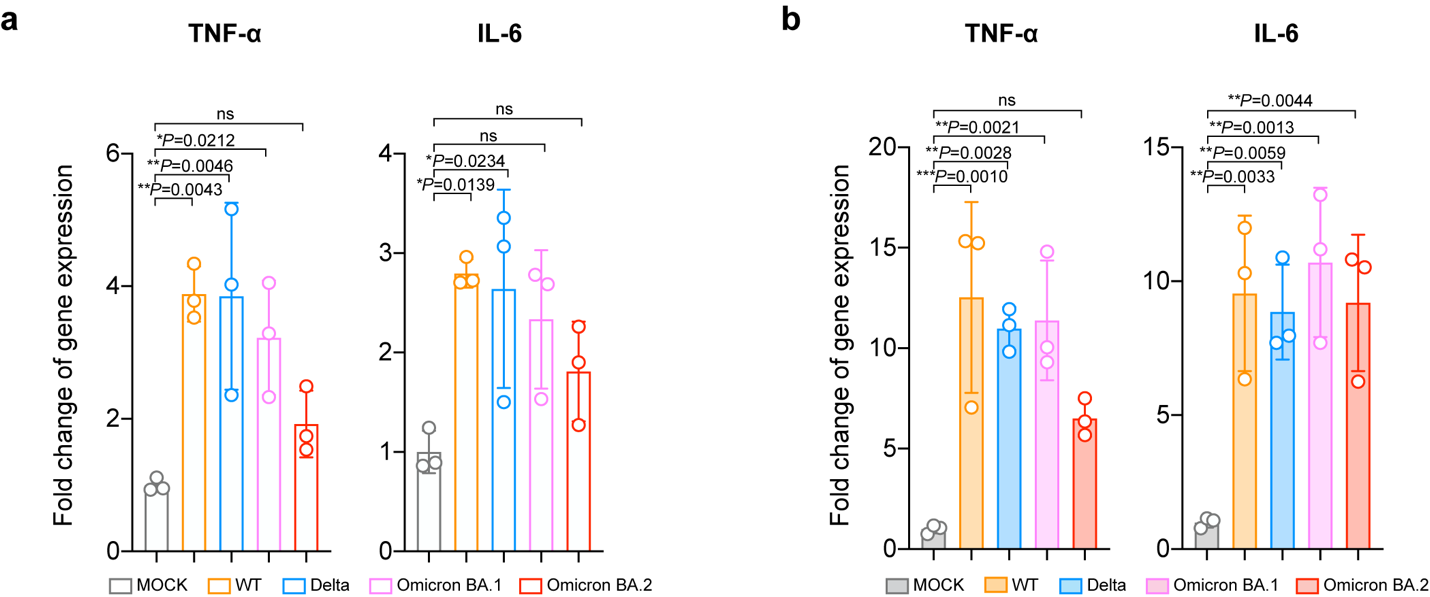
 **Figure. S3. Expression of representative pro-inflammatory genes in SARS-CoV-2-infected forebrain and midbrain organoids. a** SARS-CoV-2-infected or mock-infected forebrain organoids were collected at 96 hpi. Expression of pro-inflammatory cytokines was quantified with RT-qPCR (n=3). **b** SARS-CoV-2-infected or mock-infected midbrain organoids were collected at 96 hpi. Expression of pro-inflammatory cytokines was quantified with RT-qPCR (n=3). Data represented mean and standard deviations from the indicated number of biological repeats. Statistical significance between groups was determined with one-way ANOVA with Dunnett’s tests.

Figure. S4.

**
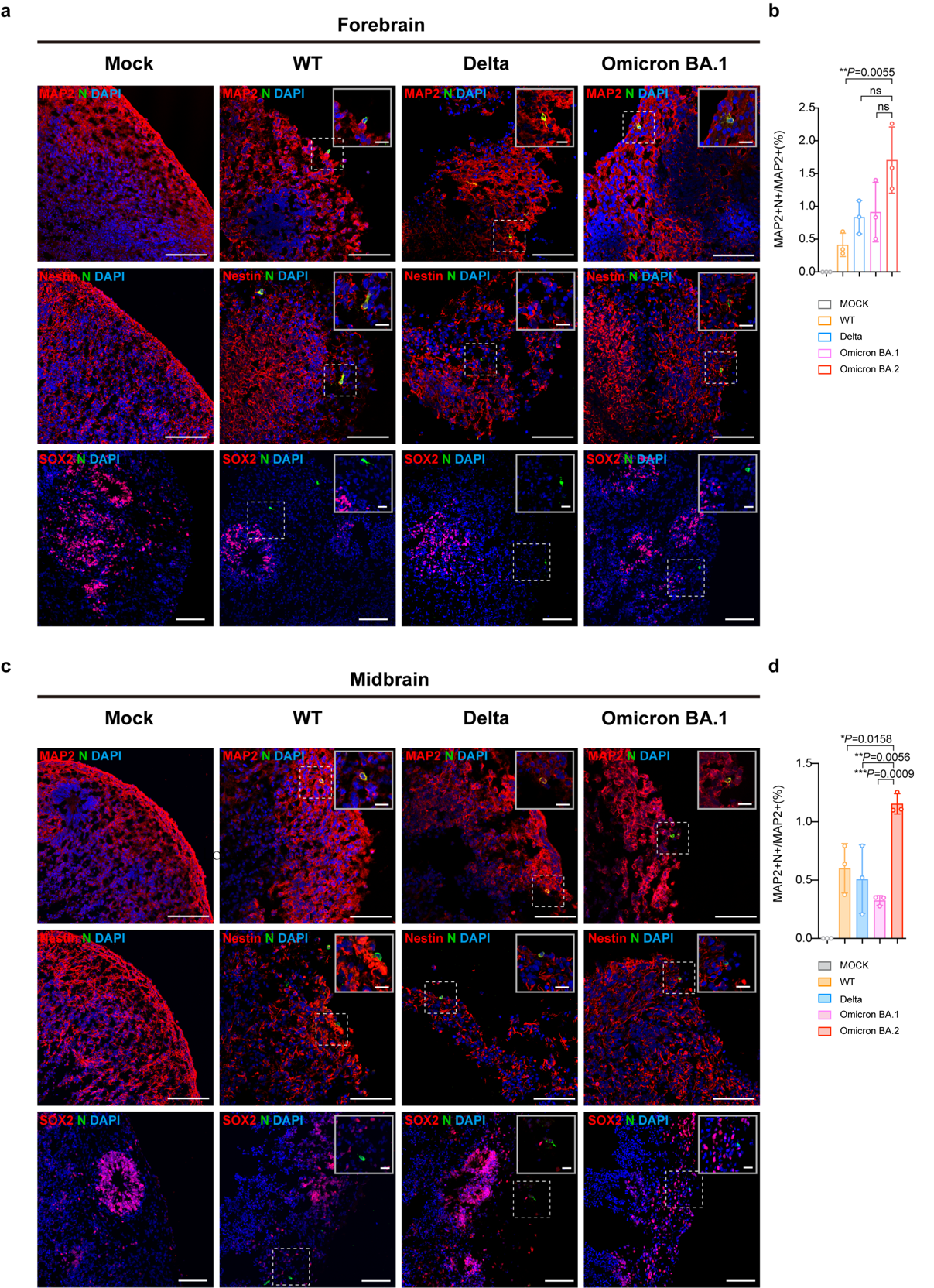
**

**Figure. S4. Neurotropism of SARS-CoV-2 in human forebrain and midbrain organoids. a** SARS-CoV-2 WT, Delta, Omicron BA.1, or mock-infected forebrain organoids were collected at 96 hpi. Colocalization between viral N protein and cellular markers (MAP2, Nestin, SOX2) was determined with immunostaining. Scale bar represents 100 μm. Scale bar in the insets represents 20 μm. **b** Quantification of MAP2+N+ neurons among MAP2+ neurons in infected forebrain organoids (n=3). **c** SARS-CoV-2 WT, Delta, Omicron BA.1, or mock-infected midbrain organoids were collected at 96 hpi. Colocalization between viral N protein and cellular markers (MAP2, Nestin, SOX2) was determined with immunostaining. Scale bar represents 100 μm. Scale bar in the insets represents 20 μm. **d** Quantification of MAP2+N+ neurons among MAP2+ neurons in infected midbrain organoids (n=3). Data represented mean and standard deviations from the indicated number of biological repeats. Statistical significance between groups was determined with one-way ANOVA with Tukey’s tests.

Figure. S5.


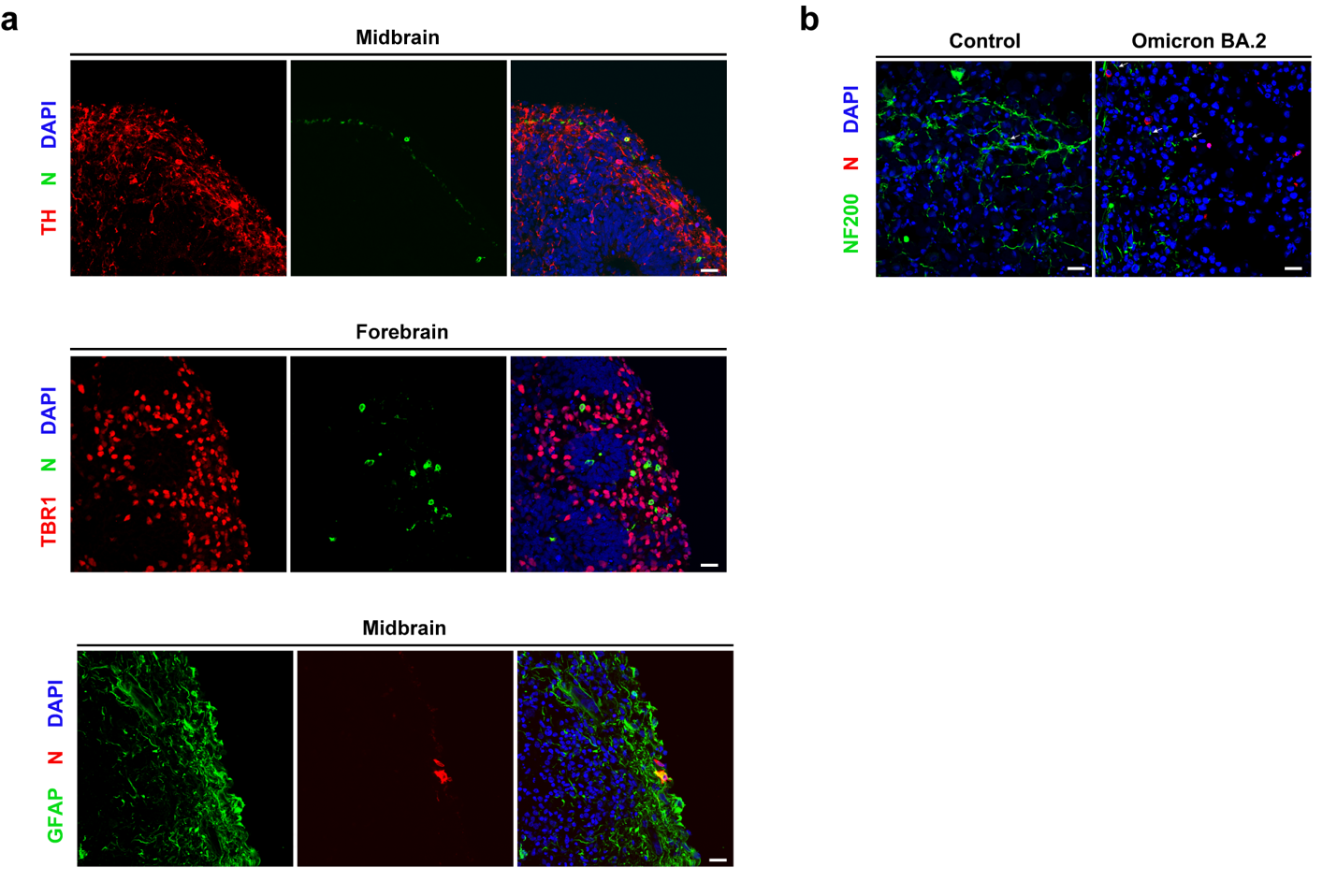


Figure. S5. Neuron tropism of Omicron BA.2. a Day 30 midbrain Omicron BA.2-infected organoids: N/TH; Day 30 forebrain Omicron BA.2-infected organoids: N/TBR1; Day 120 midbrain Omicron BA.2-infected organoids: N/GFAP. b Day 120 forebrain Omicron BA.2-infected organoids: N/NF200. Scale bar represents 20 μm.

Figure. S6.

**
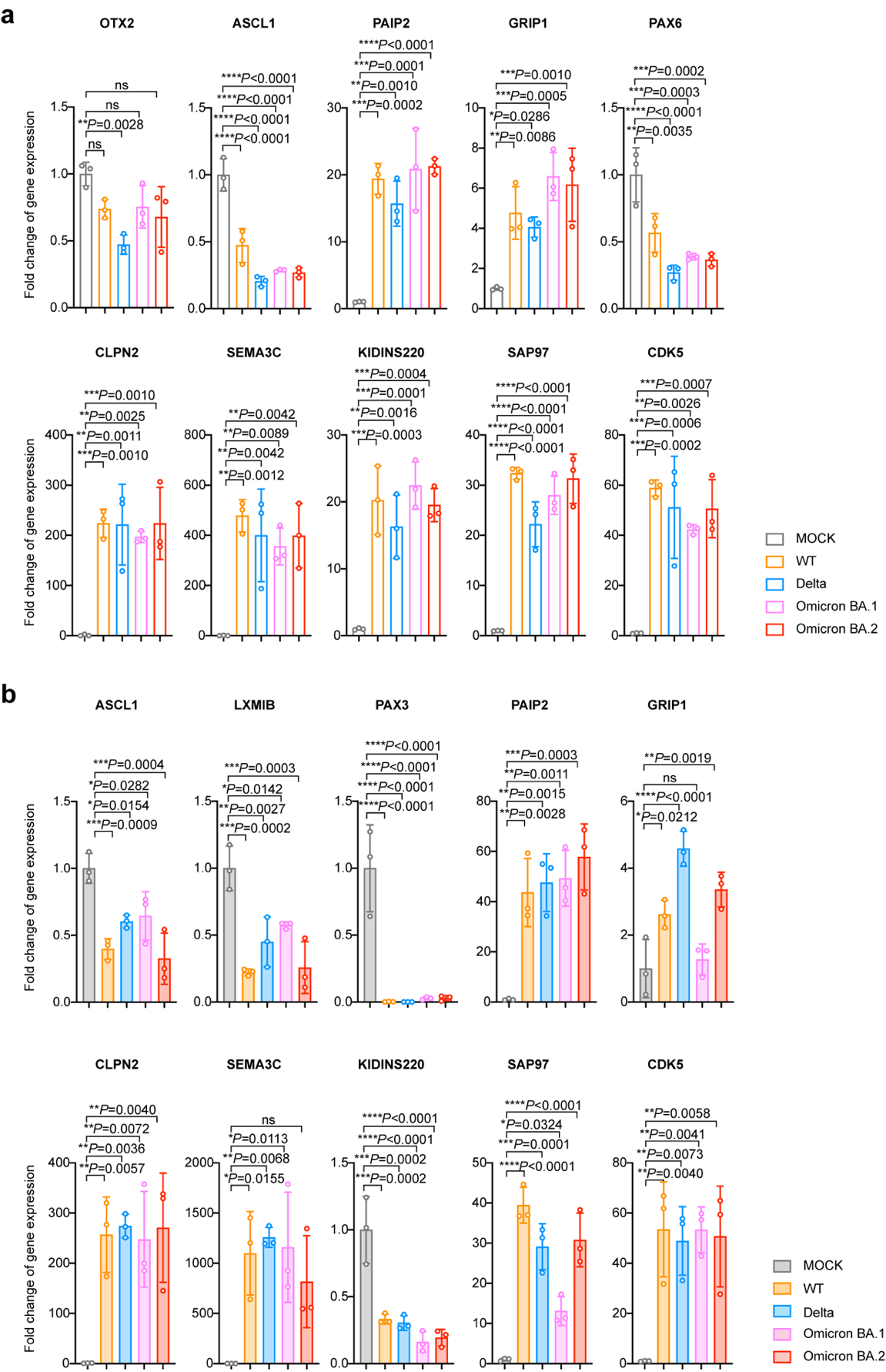
**

**Figure. S6. SARS-CoV-2 infection disrupts the expression of neural markers in human forebrain and midbrain organoids. a** SARS-CoV-2-infected or mock-infected forebrain organoids were collected at 96 hpi. Expression of neural markers was quantified with RT-qPCR (n=3). **b** SARS-CoV-2-infected or mock-infected midbrain organoids were collected at 96 hpi. Expression of neural markers was quantified with RT-qPCR (n=3). Data represented mean and standard deviations from the indicated number of biological repeats. Statistical significance between groups was determined with one-way ANOVA with Dunnett’s tests.

Figure. S7.

**
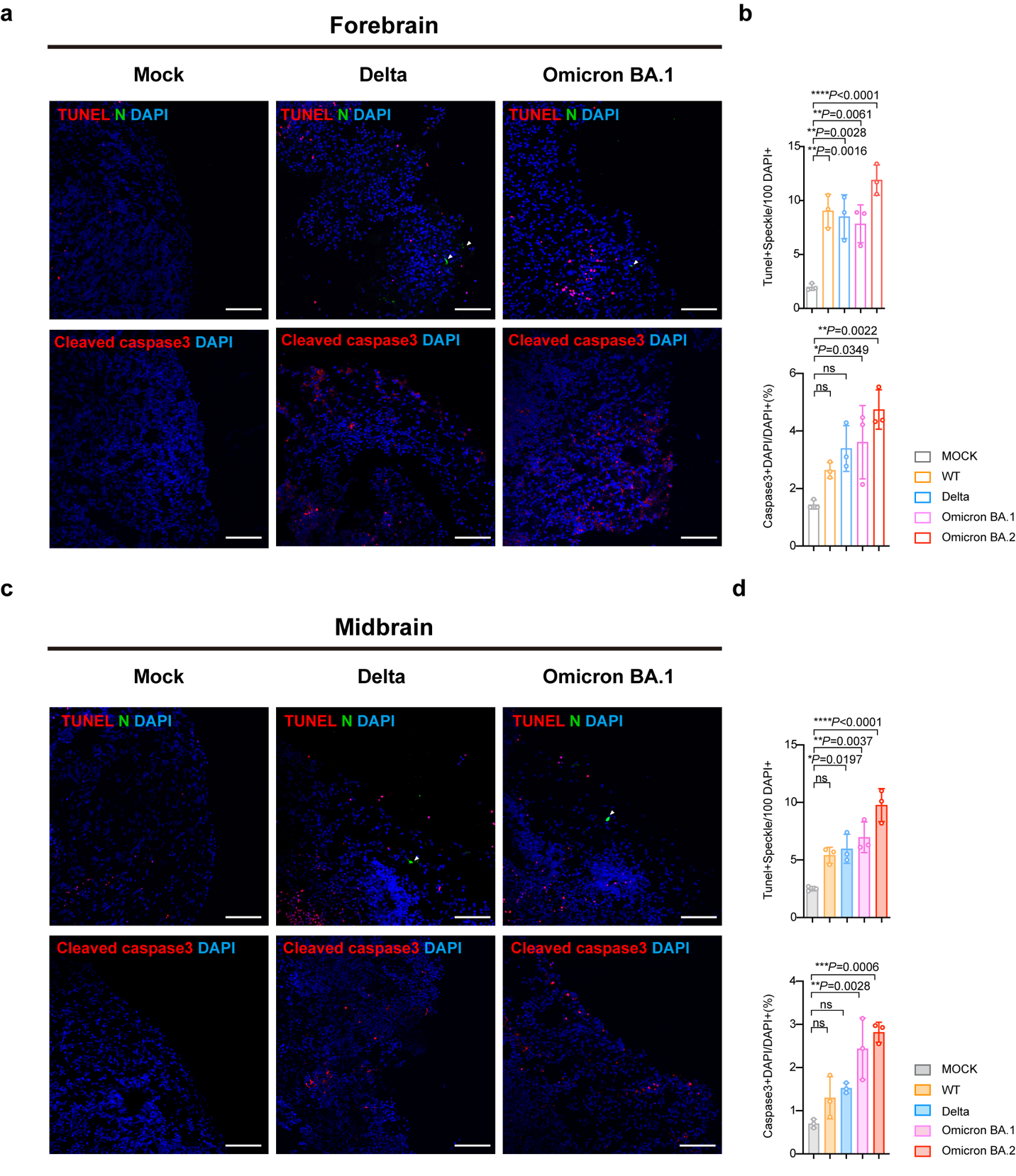
**

**Figure. S7. SARS-CoV-2 infection triggers apoptosis in human forebrain and midbrain organoids. a** SARS-CoV-2 Delta-, Omicron BA.1-, or mock-infected forebrain organoids were collected at 96 hpi. TUNEL and cleaved caspase-3 staining were performed. Scale bar represents 100 μm. **b** Quantification of TUNEL and cleaved caspase-3 staining in infected forebrain organoids (n=3). **c** SARS-CoV-2 Delta-, Omicron BA.1-, or mock-infected midbrain organoids were collected at 96 hpi. TUNEL and cleaved caspase-3 staining were performed. Scale bar represents 100 μm. **d** Quantification of TUNEL and cleaved caspase-3 staining in infected midbrain organoids (n=3). Data represented mean and standard deviations from the indicated number of biological repeats. Statistical significance between groups was determined with one-way ANOVA with Tukey’s tests.

Figure. S8.

**
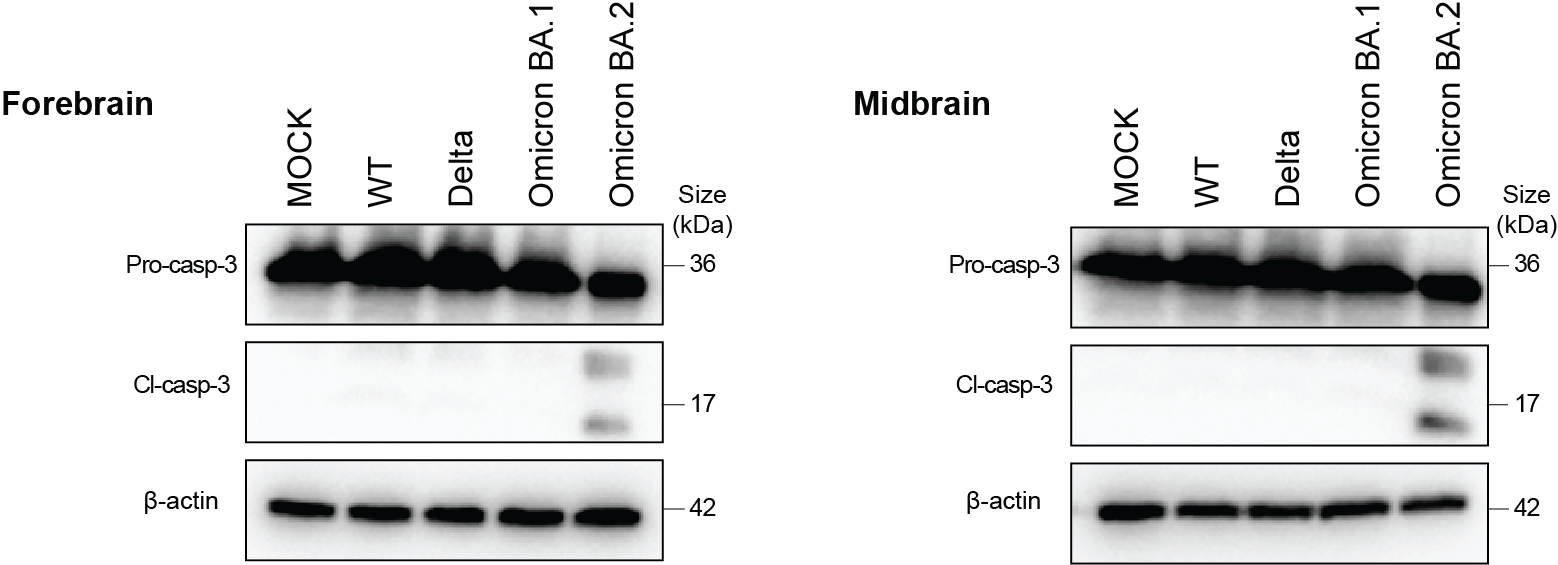
**

**Figure. S8. SARS-CoV-2 infection triggers apoptosis in human forebrain and midbrain organoids.** SARS-CoV-2 WT-, Delta-, Omicron BA.1-, Omicron BA.2-, or mock-infected forebrain and midbrain organoids were collected at 72 hpi. Expression of cleaved caspase-3 was determined with Western Blots.

Figure. S9.

**
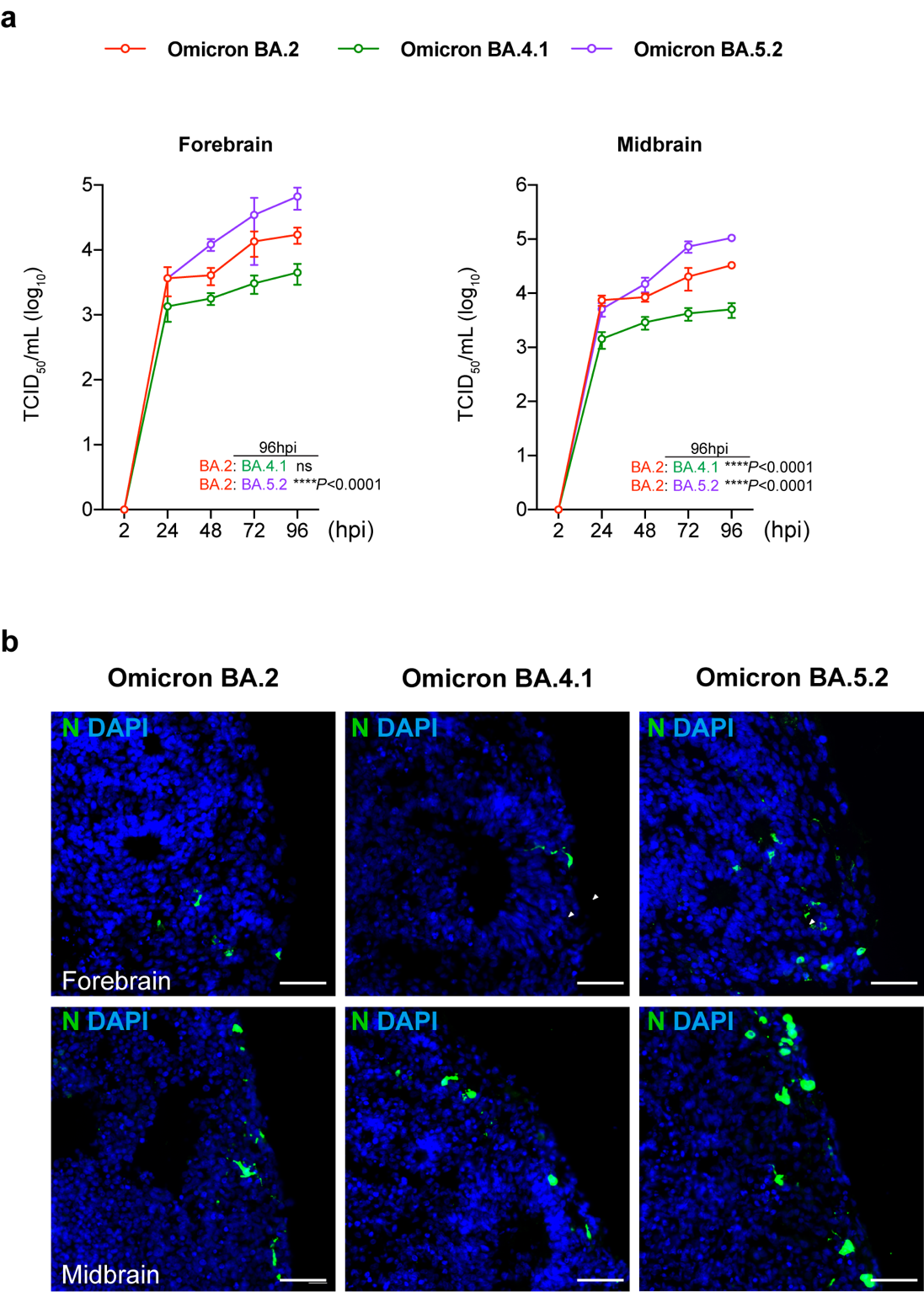
**

**Figure. S9. Replication of Omicron BA.4.1 and BA.5.2 in human embryonic stem cells-derived brain organoids. a** Human forebrain and midbrain organoids were challenged with 3x10^5^ PFU of Omicron BA.2, Omicron BA.4.1 and Omicron BA.5.2. Virus replication in supernatants was determined by TCID_50_ assays (n=3). **b** SARS-CoV-2 Omicron BA.2-, BA.4.1- and BA.5.2- infected forebrain and midbrain organoids were collected at 96 hpi. N protein was determined with immunostaining. Scale bar represents 50 μm. Data represented mean and standard deviations from the indicated number of biological repeats. Statistical significance between groups was determined with two-way ANOVA with Dunnett’s tests.

Figure. S10.


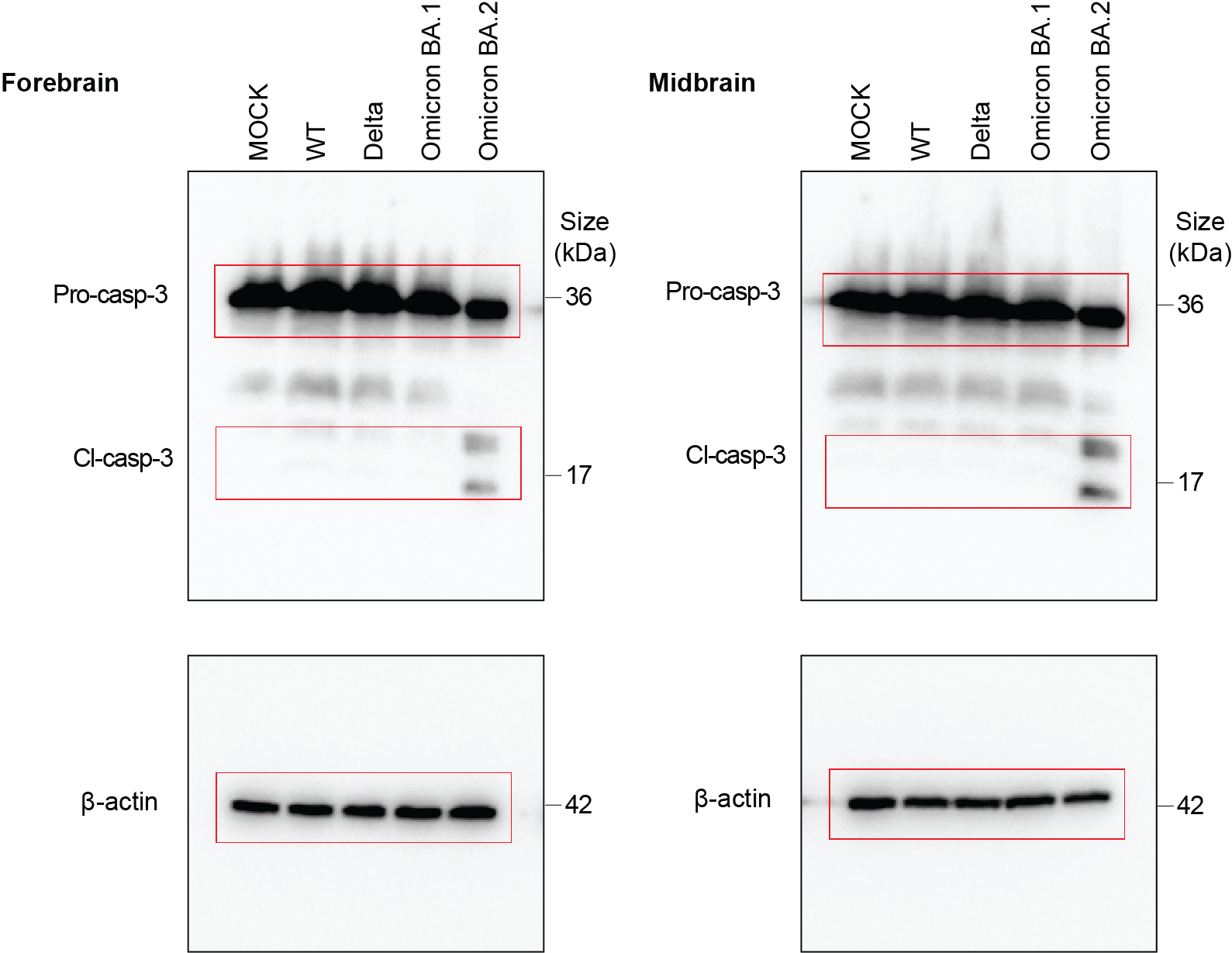


**Figure. S10. Uncropped figures of supplementary Fig. 8.** SARS-CoV-2 infection triggers apoptosis in human forebrain and midbrain organoids. SARS-CoV-2 WT-, Delta-, Omicron BA.1-, Omicron BA.2-, or mock-infected forebrain and midbrain organoids were collected at 72 hpi. Expression of cleaved caspase-3 was determined with Western Blots.

Table S1.

| Gene | Forward (5′-3′) | Reverse (5′-3′) |
| --- | --- | --- |
| SARS-CoV-2 RdRp | CGCATACAGTCTTRCAGGCT | GTGTGATGTTGAWATGACATGGTC |
|  | Probe 5’- FAM-TTAAGATGTGGTGCTTGCATACGTAGAC-lABkFQ-3’ | |
| sgE | CGATCTCTTGTAGATCTGTTCTC | ATATTGCAGCAGTACGCACACA |
| IFN-α | AGAATCACTCTCTATCTGAAAGAGAAGAAATA | TCATGATTTCTGCTCTGACAACCT |
| IFN-β | GCCGCATTGACCATCT | AGGAGTACAGTCACTGTG |
| IFN-λ1 | TATACATATGAAGCCGACTACTACTG | CCGGATCCTCAGGTGCTCTCCGGGTG |
| IL-6 | GTAGCCGCCCCACACAGACAGCC | AACCCACCAGACAAACCA |
| TNFα | CAAGGACAGCAGAGGACCAG | TGGCGTCTGAAGGTTGTTTT |
| STAT1 | CAGCTTGACTCAAAATTCCTGGA | TGAAGATTACGCTTGCTTTTCCT |
| STAT2 | CCAGCTTTACTCGCACAGC | AGCCTTGGAATCATCACTCCC |
| CAPN2 | GTGACTTCCTGAGGCACTATTC | CCTCCAGTTCCCATCCATTT |
| SEMA3A | GGATCAGCCGTGTGTATGTATAG | TTGATAAGGCACCCATTGATAGT |
| CDK5 | GACCAAGCTGCCAGACTATAAG | CTCCCTGTGGCATTGAGTTT |
| GRIP1 | GCCACAGAAACTCTCTCTTCTC | CCATCACTCTGTCTCCAATCTG |
| SAP97 | TGCACAATATCGACCTGAAGAA | AGAGAACCTGACCCTGAACTA |
| KIDINS220 | AAAGCCGAAGGGAAAGTAGAG | AGGAGCGCATCCGATAAATAC |
| PAIP2 | GAAGAGGAGTTATGGGAAGAAGAA | GGAGATCTCGAGCTGGAATAAA |
| ACE2 | CATTGGAGCAAGTGTTGGATCTT | GAGCTAATGCATGCCATTCTCA |
| TMPRSS2 | CTCTACGGACCAAACTTCATC | CCACTATTCCTTGGCTAGAGTA |

Table S1. (Continue)

| Gene | Forward (5′-3′) | Reverse (5′-3′) |
| --- | --- | --- |
| Cathepsin B | AGAGTTATGTTTACCGAGGACCT | GATGCAGATCCGGTCAGAGA |
| Cathepsin L | GTGGACATCCCTAAGCAGGA | CACAATGGTTTCTCCGGTC |
| GAPDH | ATTCCACCCATGGCAAATTC | CGCTCCTGGAAGATGGTGAT |
| CHOP | AGAACCAGGAAACGGAAACAGA | TCTCCTTCATGCGCTGCTTT |
| PUMA | CAGACTGTGAATCCTGTGCT | ACAGTATCTTACAGGCTGGG |
| SOX2 | GCTACAGCATGATGCAGGACCA | TCTGCGAGCTGGTCATGGAGTT |
| ASCL1 | GGTGATCGCACAACCTGCAT | GTTCTGAGCGCTTCCCGTTT |
| FOXG1 | GAGCGACGACGTGTTCATC | GCCGTTGTAACTCAAAGTGCTG |
| FOXA2 | GGAGCAGCTACTATGCAGAGC | CGTGTTCATGCCGTTCATCC |
| LMX1B | TTCCTGATGCGAGTCAACGAG | GCAGTACAGTTTCCGATCCCG |
| PAX3 | GGCTTTCAACCATCTCATTCCCG | GTTGAGGTCTGTGAACGGTGCT |
| OTX2 | CCCTCACTCGCCACATCTAC | GTTCAGAGTCCTTGGTGGGT |
